# Tiny guardian of the spring: Description of the first Islamiinae (Mollusca, Hydrobiidae) from the Caucasus

**DOI:** 10.3897/zookeys.1272.178534

**Published:** 2026-03-10

**Authors:** Elizaveta M. Chertoprud, Jozef Grego, Levan Mumladze

**Affiliations:** 1 Department of Invertebrate Zoology, Biological Faculty, Lomonosov Moscow State University, 1, Leninskie Gory, 119991 Moscow, Russia Biological Faculty, Lomonosov Moscow State University Moscow Russia; 2 Laboratory of Synecology, Severtsov Institute of Ecology and Evolution, Russian Academy of Sciences, Leninsky Prospect, 3, 119071, Moscow, Russia Institute of Zoology, Ilia State University Tbilisi Georgia; 3 Horná Mičiná 219, 97401 Banská Bystrica, Slovakia Severtsov Institute of Ecology and Evolution, Russian Academy of Sciences Moscow Russia; 4 Department of Biology, SubBioLab, Biotechnical Faculty, University of Ljubljana, Jamnikarjeva 101, 1000 Ljubljana, Slovenia Biotechnical Faculty, University of Ljubljana Ljubljana Slovenia; 5 Institute of Zoology, Ilia State University, Tbilisi, Kakutsa Cholokashvili Ave 3/5, Tbilisi 0162, Georgia Unaffiliated Banská Bystrica Slovakia

**Keywords:** Cave, biodiversity, endemism, freshwater snail, Georgia, groundwater, microgastropods, new genus, new species, stygobiont, taxonomy

## Abstract

*Gveleshapia kvevri***gen. et sp. nov**. represents the first formally described species of Islamiinae from the Caucasus, extending the distribution of this subfamily far beyond its traditionally outlined Mediterranean range. The new taxon was discovered during fieldwork in 2021–2022 in Mandaeti village, Imereti, Georgia. Morphological examination of seven ethanol-preserved specimens and six empty shells, combined with phylogenetic analysis, confirms its distinct taxonomic status. The genus is characterized by an elongate-conical shell with up to 5.2 whorls, trilobate penis morphology, and the absence of a bursa copulatrix in the female reproductive system. Phylogenetic reconstruction places *Gveleshapia kvevri***sp. nov**. within a highly supported clade as sister to *Islamia valvataeformis* (Möllendorff, 1873) and *Alzoniella braccoensis* Bodon & Cianfanelli, 2004. The species is known from two spring localities 1.7 km apart, confirming its critically restricted stygobiont distribution. The findings highlight the taxonomic instability of Islamiinae, which appears paraphyletic in molecular analyses; the Caucasian lineage of *Gveleshapia kvevri***sp. nov**. further underscores the need for a comprehensive systematic revision. The localized distribution and anthropogenic pressures warrant conservation consideration.

## Introduction

Representing the most diverse family of non-marine aquatic molluscs in the Caucasus, Hydrobiidae Stimpson, 1865 (sensu Wilke et al. 2013) currently includes approximately 45 species in 13 genera in this region. Based on recent studies of Caucasian fauna, the region is recognized as a global center of stygobiotic molluscan radiation (Vinarski et al. 2014; Vinarski and Palatov 2019; Grego et al. 2020; Chertoprud et al. 2020, 2021, 2023, 2025), reinforcing its status as a global biodiversity hotspot. Here, ‘stygobiont’ is used in the strict sense for obligate subterranean species unable to survive outside groundwater habitats, as evidenced by troglomorphic traits such as eye reduction and depigmentation (Culver and Pipan 2019). A recent multi-locus phylogenetic analysis (Chertoprud et al. 2025) established a revised taxonomy of Caucasian stygobiotic hydrobiids, dividing them into three subfamilies: Caucasopsiinae Chertoprud & Vinarski, 2025, Belgrandiellinae Radoman, 1983, and Islamiinae Radoman, 1973.

**Figure 1.**
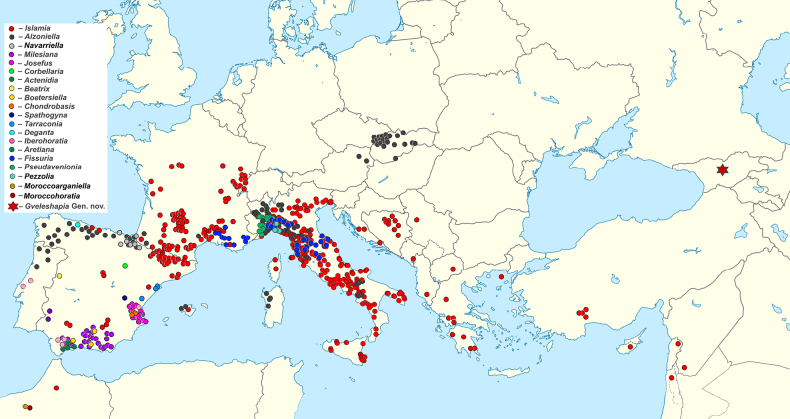
Distribution map of Islamiinae genera, based on literature data (Radoman 1983; Bertrand 2004; Arconada and Ramos 2006; Bodon et al. 2007; Glöer and Grego 2015; Glöer et al. 2015; Bodon and Cianfanelli 2022; García-Guerrero et al. 2023; Ghamizi et al. 2024). The significant distribution range extension towards Georgia is apparent.

The subfamily Islamiinae Radoman, 1973 has primarily been associated with the Mediterranean region. Fig. 1 shows a distribution map of Islamiinae genera based on literature data (Radoman 1983; Bertrand 2004; Arconada and Ramos 2006; Bodon et al. 2007; Glöer and Grego 2015; Glöer et al. 2015; Bodon and Cianfanelli 2022; García-Guerrero et al. 2023). Its type genus, *Islamia* Radoman, 1973, is distributed from the Iberian Peninsula (Arconada and Ramos 2006) through southern France (Bertrand 2004) and Italy (Bodon et al. 2001) to the Balkans (Radoman 1983; Glöer and Grego 2015; Glöer et al. 2015; Reischütz et al. 2018), encompassing a high diversity of 56 recent and seven fossil stygobiotic, stygophilic, and rarely crenobiotic species. The Iberian Peninsula represents a hotspot for Islamiinae diversity (Arconada and Ramos 2006), hosting numerous endemic genera such as *Actenidia* Arconada & Ramos, 2024; *Aretiana* Delicado & Ramos, 2021; *Beatrix* J. P. Miller, García-Guerrero, Delicado & Ramos, 2024; *Boetersiella* Arconada & Ramos, 2001; *Chondrobasis* Arconada & Ramos, 2001; *Corbellaria* Girardi & Boeters, 2012; *Deganta* Arconada & Ramos, 2019; *Iberhoratia* Arconada & Ramos, 2007; *Josefus* Arconada & Ramos, 2006; *Milesiana* Arconada & Ramos, 2006; *Navarriella* Boeters, 2000 and *Spathogyna* Arconada & Ramos, 2002. The genus *Alzoniella*, with a wide distribution from the Iberian Peninsula (Rolán and Boeters 2015; García-Guerrero et al. 2023), through southern France (Bertrand 2004), Italy (Bodon and Cianfanelli 2022), northward and with penetration to the eastern Alps (Reischütz 1983) and westward to the Carpathians (Ložek and Brtek 1964; Beran and Horsák 2001; Szarowska et al. 2011), appears polyphyletic (Delicado et al. 2023). Recent molecular studies have expanded the subfamily to include additional Iberian and Apennine genera like *Tarraconia* Ramos & Arconada, 2000, *Pseudavenionia* Bodon & Giusti, 1982, and *Pezzolia* Bodon & Giusti, 1986, while excluding others such as *Hauffenia* Pollonera, 1898 (Delicado et al., 2023) and *Avenionia* (Nicolas, 1882) as well as the Moroccan genera *Maroccoarganiella* Ghamizi & Falniowski, 2024 and *Maroccohoratia* Ghamizi & Falniowski, 2024 (Ghamizi et al. 2024). Some *Islamia* species have been reported from western Turkey (*Islamia anatolica* Radoman, 1973; *Islamia bunarbasa* (Schütt, 1964); *Islamia burdurica* Yıldırım, Çağlan Kaya, Gürlek & Koca, 2017; *Islamia pseudorientalica* Radoman, 1973), Cyprus (*Islamia mylonas* Radea, Parmakelis, Demetropoulos & Vardinoyannis, 2017), Israel (*Islamia mienisi* (Schütt, 1991)), Syria and Lebanon (*Islamia gaillardoti* (Germain, 1911)) and Morocco (*Islamia karawiyiensis* Mabrouki, Glöer & Taybi, 2021). However, given the frequent morphological convergences in truncatelloids gastropods (Falniowski 2022; Grego et al. 2025), these assignments, based primarily on shell and occasionally genital morphology, must be considered provisional pending molecular confirmation. Furthermore, the most recent phylogenetic studies indicate that Islamiinae is a paraphyletic group (Delicado et al. 2023; Chertoprud et al. 2025), requiring future reclassification.

In the Caucasus, representatives of Islamiinae had not been recorded until Chertoprud et al. (2025), who reported an unknown taxon within the Islamiinae clade as Gen. 5 sp. Here, we provide a taxonomic description of this new genus and species as *Gveleshapia kvevri* gen. et sp. nov., based on its shell morphology and anatomy. Furthermore, we use the molecular phylogenetic context from Chertoprud et al. (2025) to establish its taxonomic position and discuss the biogeographical implications for Islamiinae.

## Materials and methods

### Sample collection

Material for this study was collected during the authors’ fieldwork in 2021–2022. We collected the snails by hand or by sieving sediments, following the protocol of Grego et al. (2017). Details on the sampled localities and voucher specimens are provided in the section ‘Systematics’.

### Morphological study

A morpho-anatomical study was conducted on seven ethanol-preserved specimens and six empty shells (see Suppl. material 4). Images of the shells were obtained using a Carton SPZT50 microscope equipped with a TOUPCAM U3CMOS camera and a LeicaM165C stereo microscope with a digital camera Leica DFC420 (5.0MP). ImageJ image analysis software (Rueden et al. 2017) was used to measure the specimens. Shell morphology was described following the terminology of Davis et al. (1992) and Hershler and Ponder (1998). Ten linear measurements were taken from six empty shells and five ethanol-preserved specimens (*N* = 11) following the scheme given in Anistratenko et al. (2022) (see fig. 3 in Anistratenko et al. 2022); for specimen voucher details see Suppl. material 5. We also counted the whorl number using the method employed in our previous studies (see Chertoprud et al. 2023). The dissections were performed under a stereoscopic microscope in an ethanol-filled Petri dish with a black plasticine bottom. Using forceps and minute pins, the body was freed from the shell and the mantle cavity was dissected by a micro scalpel. For radular morphology examination, the buccal bulbs of two specimens were extracted and dissolved with a 3% solution of sodium hypochlorite (NaClO). The radula was isolated from the buccal mass and observed under a stereomicroscope. When only the radula remained, the hypochlorite was carefully removed using a capillary pipette, and the radula was kept in distillate, which was changed about five times. The same technique was used for operculum isolation. The radulae were transferred into a drop of distilled water onto a glass slide, and then oriented and dried. The shells were cleaned using hypochlorite for five to ten minutes, then in distilled water for one to two hours. Radulae, opercula, shells and protoconchs were mounted on stubs, sputter-coated with gold ions in vacuum and imaged on Vega 3 Tescan electronic microscope in the Y.A. Orlov Paleontological Museum at the Paleontological Institute of the Russian Academy of Sciences (Moscow). Morphological characteristics of the soft body, including the ctenidium, osphradium and genitalia of males and females were examined using the terminology of Hershler and Ponder (1998).

The type material of the newly described species has been deposited in the collection of the Ilia State University, Tbilisi, Georgia (ISU). Additional paratypes are housed in the authors’ personal research collections (J. Grego, Banská Bystrica, Slovakia).

Abbreviations used in the text:

**SH** Shell height;

**SW** Shell width;

**BWH** Body whorl height;

**BWW** Body whorl width;

**SpH** Spire height;

**AH** Aperture height;

**AW** Aperture width;

**WN** Whorl number;

**sr** seminal receptacle;

**ISU** Ilia State University, Tbilisi, Georgia;

**EC** Elizaveta Chertoprud;

**JG** Jozef Grego.

## Results

### Systematics

**Class Gastropoda Cuvier, 1795**

**Subclass Caenogastropoda Cox, 1960**

**Order Littorinimorpha Golikov & Starobogatov, 1975**

**Superfamily Truncatelloidea Gray, 1840**

**Family Hydrobiidae W. Stimpson, 1865**

**Subfamily Islamiinae Radoman, 1973**

#### Gveleshapia gen. nov.

Taxon classificationAnimaliaLittorinimorphaHydrobiidae

C046A2BF-ABE8-506F-A2A3-5FD449A5A3F9

https://zoobank.org/B450FBF9-40DE-429F-BF1E-A6959AE9E57C

##### Type species.

*Gveleshapia kvevri* sp. nov.

##### Etymology.

The genus is named after the Gveleshapi (გველეშაპი), a mighty dragon or serpent from Georgian mythology that was believed to dwell in deep rivers, lakes, and springs or even own water sources, demanding sacrifices in exchange for water. Feminine gender.

##### Diagnosis.

Shell shape conic, with five whorls; protoconch clearly bordered (by axial growth line fold), covered by irregular honeycomb-like pits and folds of variable density; operculum ovate, thin and flat, paucispiral with submarginal nucleus, inner side of nucleus area thickened; eyes absent, unpigmented; ctenidium contain nine filaments; osphradium elongate-ovate; central radular tooth formula 5-1-5/1-1; lateral radular tooth formula (4-5)-1-(4-5); pallial gland complex of female reproductive organs 4–5 times longer than wide; bursa copulatrix absent; capsule gland up to 2/3 length of pallial gland complex; seminal receptacle small, up to 1/4 length of coiled part of oviduct, elongate-pyriform; renal oviduct U-shaped with evenly thickened walls; penis trilobate, gradually tapering, with two medial lobes, dilated along left distal-medial edge; prostate bean-shaped.

##### Differential diagnosis.

Conchologically, *Gveleshapia* gen. nov. differs from most Islamiinae genera in possessing a distinctly conical shell with a ribbed surface sculpture. In contrast, the majority of Islamiinae genera (e.g., *Islamia*, *Josefus*, *Iberhoratia*, *Hauffenia*, *Beatrix*, *Actenidia*) exhibit valvatoid, planorboid, ovate-conic, or broadly conical shells lacking such pronounced ribbing. The male reproductive system provides additional diagnostic features. The penis in *Gveleshapia* gen. nov. is distinctly trilobate, with a well-developed multilobate structure that differs in configuration from the penial morphology observed in Caucasian hydrobiid genera such as *Caucasopsis*, *Schapsugia*, and *Sitnikovia*, as well as from other described Islamiinae taxa. The female reproductive system is particularly distinctive. *Gveleshapia* gen. nov. lacks a bursa copulatrix, a condition that is rare among Islamiinae in which female genitalia have been described. In addition, the oviduct is strongly proximally displaced relative to the albumen gland, resulting in a lateral positioning of the seminal receptacle adjacent to the shell wall. Although a lateral loop of the oviduct is common in hydrobiids, the degree of displacement observed in *Gveleshapia* gen. nov. is pronounced. This unique combination of (1) a conical ribbed shell, (2) a distinctly trilobate penis, and (3) a female reproductive system lacking a bursa copulatrix and exhibiting a strongly displaced oviduct clearly separates *Gveleshapia* gen. nov. from all previously described Islamiinae genera.

##### Species included.

*Gveleshapia kvevri* sp. nov.

##### Molecular data.

The new genus forms a highly supported clade (PP = 1, BS = 100) (Chertoprud et al. 2025; see also Suppl. materials 1–3).

##### Distribution.

The genus is known only from the groundwater of the Zemo Imereti karst plateau, specifically from springs near Mandaeti village.

#### Gveleshapia kvevri sp. nov.

Taxon classificationAnimaliaLittorinimorphaHydrobiidae

B5BF9640-514B-571F-A43E-9628688A5D6D

https://zoobank.org/F99C91A2-2B13-41F7-BAC7-8A0AD3E2DFE9

Figs 3, 4, 5

##### Type material.

***Holotype*: Georgia** • (♀dissected, voucher G21_I_3_2; in ethanol 90%) (Fig. 3a); Zemo Imereti, Mandaeti, Kvevri spring near the road to Tkemlovani; 42.190645°N, 43.315631°E; 700 m a.s.l.; leg. J. Grego, L. Mumladze and M. Szekeres leg.; 09. 10. 2021; deposited at Institute of Zoology, Ilia State University, Tbilisi, Georgia under repository number: ISU FM-T024-H. ***Paratypes*: Georgia** • two dry and four alcohol preserved specimens; Kvevri spring near the road to Tkemlovani, Mandaeti, Zemo Imereti, Georgia; 42.190645°N, 43.315631°E; 700 m a.s.l.; leg. J. Grego, L. Mumladze and M. Szekeres leg.; 09. 10. 2021; (♀ dissected, G21_I_3_3; ♂ dissected, G21_I_3_4) 2 alcohol preserved specimens on coll. ISU FM-T024-P1/P2; 2 dry and two alcohol preserved specimens on coll. JG F1920. • two dry specimens; spring-sourced water reservoir in a small cave with pipes; vill. Mandaeti, Zemo Imereti; 42.186691°N, 43.335566°E; 740 m a.s.l.; E. Chertoprud, J. Grego, L. Mumladze, M. Olšavský and L. Vlček leg.; 27.04.2025; on coll. JG F4331. • two dry specimens; Kvevri spring near the road to Tkemlovani, Mandaeti, Zemo Imereti, Georgia; 42.190645°N, 43.315631°E; 700 m a.s.l.; J. Grego, L. Mumladze, E. Chertoprud leg.; 13. 09. 2022; on coll. JG F2155.

##### Other material examined.

**Georgia** • six dry and one alcohol preserved specimens; spring-sourced water reservoir in a small cave with pipes; vill. Mandaeti, Zemo Imereti; 42.186691°N, 43.335566°E; 740 m a.s.l.; E. Chertoprud leg.; 22 August 2021; one ♀ dissected alcohol preserved specimen in Moscow on coll. EC G21_ZI_1_1; six dry specimens in Moscow on coll. EC G21ZI_1. • two alcohol preserved specimens; Kvevri spring near the road to Tkemlovani, Mandaeti, Zemo Imereti, Georgia; 42.190645°N, 43.315631°E; 700 m a.s.l.; J. Grego, L. Mumladze, E. Chertoprud leg.; 13. 09. 2022; in Moscow on coll. EC G22I_1. • one alcohol preserved specimen; Kvevri spring near the road to Tkemlovani, Mandaeti, Zemo Imereti, Georgia; 42.190645°N, 43.315631°E; 700 m a.s.l.; leg. J. Grego, L. Mumladze and M. Szekeres leg.; 09. 10. 2021; in Moscow on coll. EC G21_I_3_1.

##### Etymology.

**Figure 2.**
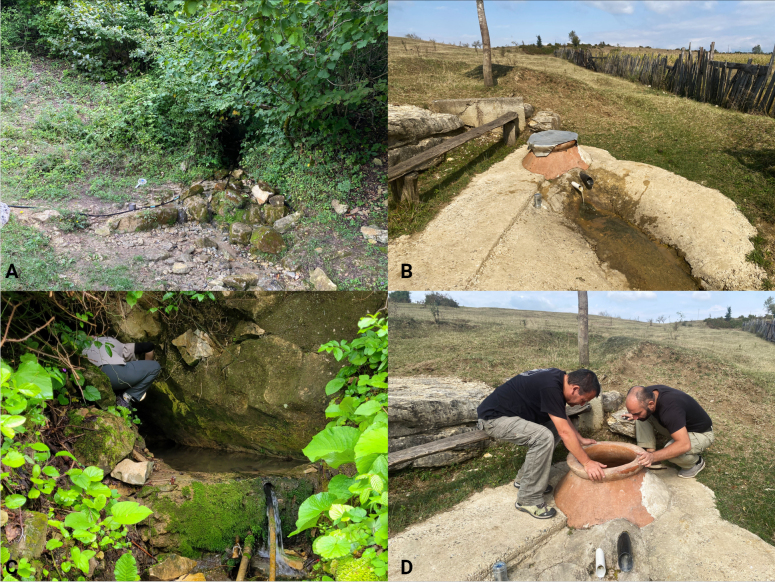
Studied localities. **A, C**. Spring-sourced water reservoir with pipes, Mandaeti, Imereti, Georgia; **B, D**. Kvevri spring near the road to Tkemlovani, Mandaeti, Imereti, Georgia (type locality).

Name derived from one of the two localities with spring water emerging from a large ceramic jar named kvevri (ქვევრი) (Fig. 2B, D), used for the fermentation, storage and aging of the traditional Georgian wine. The name also refers to the oldest archaeological evidence of winemaking traditions in the world (Neolithic, 6000–5800 BC, Gadachrili Gora, Georgia (McGovern et al. 2017), which are unambiguously associated with the use of kvevris in ancient Georgia or karasis in Armenia.

##### Description.

Measurements of holotype: SH = 2.15 mm; SW = 0.92 mm; BWH = 1.22 mm; BWW = 0.82 mm; SpH = 0.93 mm; AH = 0.64 mm; AW = 0.50 mm; WN = 4.6.

**Figure 3.**
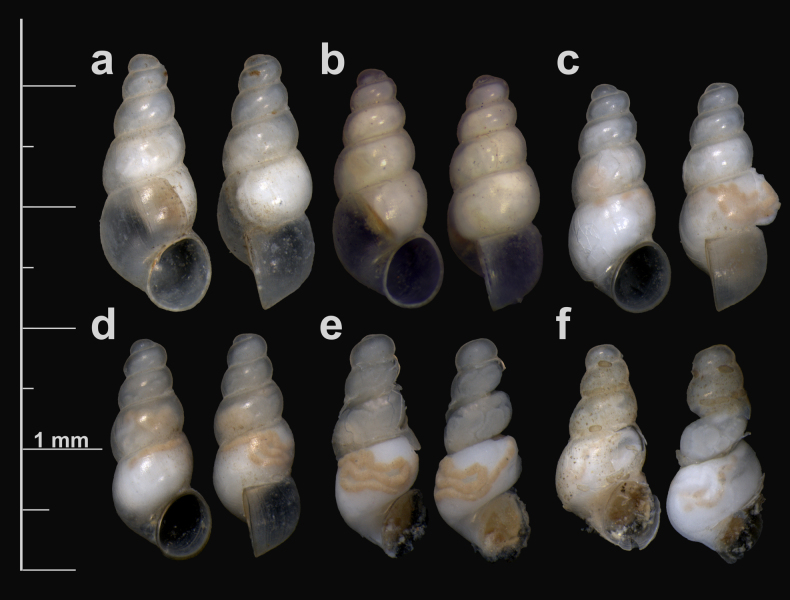
*Gveleshapia kvevri* sp. nov. **a**. G21_I_3_2f (holotype); **b**. G21_ZI_1_1f; **c**. G21_I_3_1m; **d**. G21_I_3_4m; **e**. G21_I_3_4m; **f**. G_21_I_3_3f.

**Figure 4.**
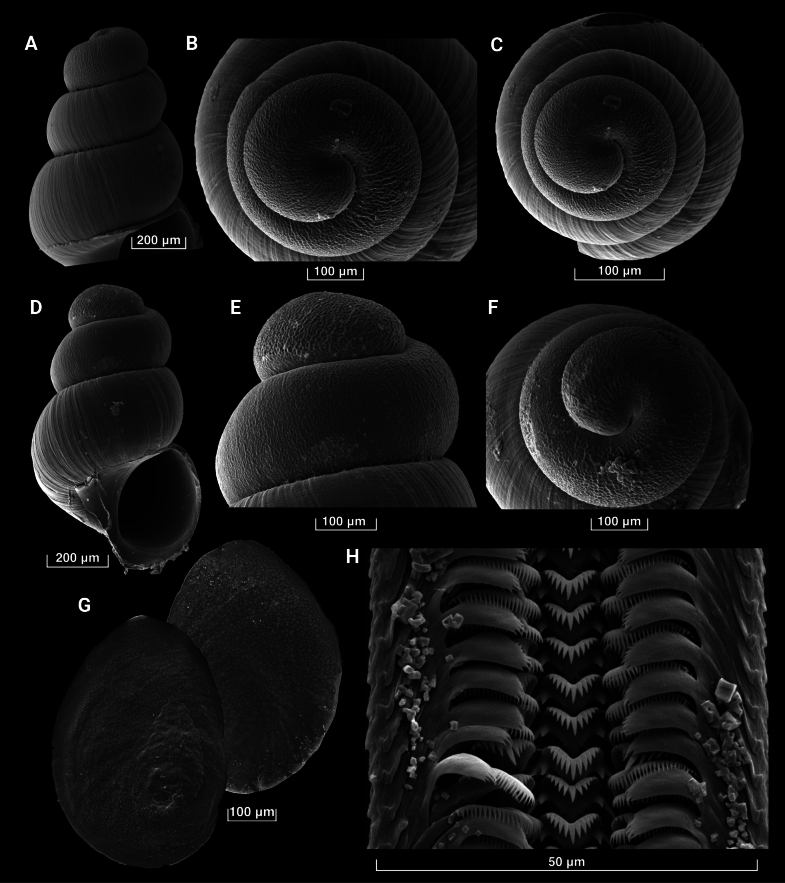
SEM photographs of *Gveleshapia kvevri* sp. nov. **A, D**. Shell apex; **B, C, F**. Protoconch, apical view; **E**. Protoconch, lateral view; **G**. Operculum; **H**. Radula. (**A–C, H**. G21_ZI_1_1; **D, E**. G22_I_1_1; **F**. G21_I_3_3; **G**. G21_I_3_2 (holotype) and G21_I_3_3.)

***Shell***. Elongate-conic, whorls number up to 5.2, shell height up to 2.15 mm, width 0.79–0.99 mm (Fig. 3; Suppl. material 5), transparent; protoconch with 1.6 whorls; protoconch with irregular pits, wrinkles and folds, well bordered by axial growth line (Fig. 4A–C); teleoconch whorls convex, separated by a deep suture, ornamented with fine axial growth lines, crossed by very weak spiral threads, resulting in an extremely delicate reticulate sculpture (Fig. 4A–E); body whorl occupies about 60% of total shell height; aperture about 30% of total shell height, ovate; aperture margin straight, upper part of inner lip touching the shell wall; umbilicus thin, slit-like or almost closed. See Suppl. material 5 for the shell measurements.

***Operculum***. Ovate, thin and flat, paucispiral with submarginal nucleus, translucent, orange or yellowish, muscle attachment located near the nucleus (Fig. 4G).

***Radula***. About 400 µm long, containing 70–80 rows of teeth. Central tooth formula 5-1-5/1-1, central cusp V-shaped, basal tongue V-shaped (Fig. 4H); lateral tooth formula (4-5)-1-(4-5), central cusp V-shaped; inner marginal teeth having ≥ 20 cusps; outer marginal teeth having ≥ 15 cusps.

**Figure 5.**
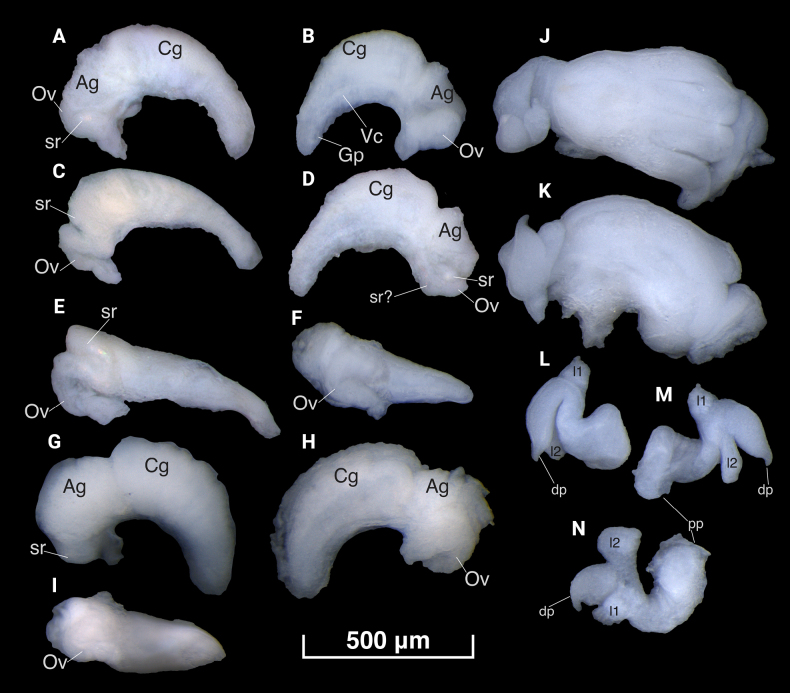
Female (**A–I**) and male (**J–N**) genitals of *Gveleshapia kvevri* sp. nov. **J, K**. Head of snail with penis attached behind it; **L–N**. Penis. Symbols for female genitals: Cg – capsule gland; Ag – albumen gland; Ov – coiled oviduct; Vc – ventral channel; Gp – genital pore; sr – seminal receptacle; sr? – another seminal receptacle (difficult to identify as it tightly lies over a widened part of the renal oviduct); dp – distal penial part; pp – proximal penial part; l1, l2 – lobes of the penis. (**A–F**. G21_I_3_2f (holotype); **G–I**. G21_ZI_1_1f; **J–M**. G21_I_3_1m; **N**. G21_I_3_4m.)

***Anatomy***. Soft body not pigmented, white; eye spots are absent (Fig. 5J–M); ctenidium with nine filaments; osphradium elongated oval.

***Male reproductive organs***. Penis trilobate, tightly folded, unpigmented, gradually tapering, with two medial lobes extending from ventral side of penis; distal portion slightly curved; basal portion slightly expanded, attached behind right tentacle (Fig. 5J–N); vas deferens runs along right penial edge; prostate gland large, bean-shaped.

***Female reproductive organs***. Capsule gland two times longer than albumen gland, occupying 2/3 of entire distal female genitalia (Fig. 5A, G), with a distinctly lobed internal structure; bursa copulatrix absent; renal oviduct is posterior to albumen gland (Fig. 5A, C, E), simple, U-shaped coiled, with one loop; unevenly widened; sr medium, elongate-pyriform, positioned on right side of albumen gland, closely adjoin oviduct loop (Fig. 5C–E); ventral channel thickened; (Fig. 5B, H); genital pore elongate-pyriform.

##### Remarks.

Phylogenetic analyses (both maximum likelihood and Bayesian inference; Suppl. materials 1–3) show all *Gveleshapia kvevri* sp. nov. specimens forming a single, highly supported clade that is sister to Islamiinae s. str. (PP = 1.00, BS = 100%). Islamiinae s. str. refers to the core, monophyletic clade on both trees (Suppl. material 1) containing *Islamia* and closely related genera (e.g., *Islamia valvataeformis* clade), excluding distantly related lineages. However, these sister-group relationships lack robust support, and Islamiinae s. l. – the broader, originally defined group including all nominal genera – appears polyphyletic in both reconstructions, indicating a need for further revision of this group (also see fig. 2 in Chertoprud et al. 2025). We therefore preliminarily assign the new genus and species to Islamiinae based on morphological criteria.

*Gveleshapia kvevri* sp. nov. shares key features with Islamiinae members, notably the multilobate penis morphology (often bearing more than one lobe in this group). The new species differs from most other species in the Islamiinae subfamily by its conical-shaped shell. Most species in this subfamily have a valvatoid, planorboid or ovate-conic shell (*Islamia*, *Josefus*, *Fissuria* Boeters, 1981, *Iberhoratia*, *Hauffenia*, *Beatrix*, *Actenidia*, etc.). This species is also distinguished by the absence of a bursa copulatrix in the female reproductive system, which is generally uncommon among those Islamiinae for which female genitalia have been described. In terms of the configuration of the oviduct and seminal receptacles, the new species’ pallial female reproductive organs are most similar to those of *Iberhoratia morenoi* Arconada, Delicado & M. A. Ramos, 2007, which is known from Spain. However, the species differ in other respects, such as shell shape, penis and the presence or absence of eyes and bursa copulatrix. It is also notable that the new species is characterized by a strong proximal displacement of the oviduct relative to the albumen gland. This results in the seminal receptacle being located on the side of the pallial gland complex that is adjacent to the shell wall (Fig. 5A). In hydrobiids, a lateral loop of the oviduct relative to the albumen gland is common (Hershler and Ponder 1998). The presence of a second seminal receptacle remains debatable. The removal of a section of the oviduct loop revealed a dilated duct section with opalescent content (Fig. 5D). It is unclear whether this structure is an adherent seminal receptacle or if sperm are stored directly within the oviduct itself. *Gveleshapia kvevri* sp. nov. is conchologically similar to representatives of other hydrobiid genera known from the Caucasian region (*Caucasopsis* Grego & Mumladze, 2020, *Schapsugia* Chertoprud, Palatov & Vinarski, 2021, *Sitnikovia* Chertoprud, Palatov & Vinarski, 2020). However, it differs significantly in the morphology of the trilobate penis, as well as in the morphology of the female genitalia, which lack a bursa copulatrix, and whose oviduct is strongly displaced beyond the albumen gland.

##### Habitat and distribution.

*Gveleshapia kvevri* sp. nov. is found in two springs in Mandaeti village (Imereti, Georgia) (Fig. 2), located 1700 m apart. The type locality is a short, 5 m-long cave spring with an artificial tap water collector that emerges from Cretaceous limestone beds. The second known locality is a small, permanent rheocrene spring emerging from Middle Miocene sandstones subjacent to Quaternary clay deposits. Both localities are hydrologically connected, emerging from the same aquifer system within a single limestone hill covering an area of only about 2 km^2^. However, the potential extent of adjacent groundwater aquifers as additional habitats cannot be assessed due to the lack of detailed hydrogeological data for the springs and the surrounding region. Live specimens were found washed out of their stygobiont habitat in fine sediments of the springhead and occasionally also attached to pieces of decaying wood. Despite extensive sampling over several years, only seven live specimens (two juveniles, five adults) were collected across both localities (all individuals were used for morphological and molecular analyses), confirming the collection sites as margins of underground populations. Most living mollusks were collected from the type locality using the bottom-sediment sieving technique (Grego et al. 2017). We also collected materials from several other springs in the vicinity, but with no further records of *G. kvevri* sp. nov. Thus far, the species is only known from two closely localized springs, and its distribution range is below two square kilometers. Both localities and the spring drainage areas are within the boundaries of villages under significant anthropogenic influence (such as overgrazing, agricultural practice, and pollution). The fact that the groundwater systems of Zemo Imereti were heavily altered by manganese mining during the 20^th^ century and are steadily exposed to pollution from a densely populated rural region (Lezhava et al. 2017), we can assume that the distribution range of the new genus could once have been much larger than the recent record would suggest. Therefore, the populations of *G. kvevri* sp. nov. and, most probably, the whole species can be considered Near Threatened.

## Discussion

The description of *Gveleshapia kvevri* sp. nov. in Georgia represents a significant northeastward range extension for the subfamily Islamiinae, which was previously considered to be predominantly Mediterranean. Phylogenetic analysis places *G. kvevri* sp. nov. within a highly supported clade (PP = 1, BS = 100) as a sister to species such as *Islamia valvataeformis* and *Alzoniella braccoensis* (Chertoprud et al. 2025). This placement is notable given its distinct elongate-conical shell, contrasting with the valvatoid or planorboid shells typical of most Islamiinae (e.g., *Islamia*, *Josefus*, *Fissuria*), and the absence of a bursa copulatrix in the female genitalia.

The discovery of this geographically isolated and morphologically distinct lineage in the Caucasus may suggest it as a descendant of a mid-to-early Miocene radiation (i.e., related to uprising of the Caucasus Mountains). This hypothesis is further supported by its basal position within the sampled Islamiinae taxa in phylogenetic reconstructions (Chertoprud et al. 2025), although Islamiinae is recovered as paraphyletic overall (Delicado et al. 2023). Nevertheless, further study, such as time-calibrated phylogenetic reconstruction, is needed to fully unravel the history of this enigmatic lineage. The current phylogeny suggests that the group’s taxonomy is unstable. The inclusion of this new Caucasian genus further underscores the need for a comprehensive systematic revision of the subfamily, which may also incorporate some additional Caucasian, Anatolian, Levantine or North African genera in the future.

## Supplementary Material

XML Treatment for Gveleshapia

XML Treatment for Gveleshapia kvevri

## Additional information

### Use of AI

No use of AI was reported.

### Author ORCIDs

Elizaveta M. Chertoprud https://orcid.org/0000-0003-0396-7463

Jozef Grego https://orcid.org/0000-0002-4977-0415

Levan Mumladze https://orcid.org/0000-0002-2172-6973

### Data availability

All of the data that support the findings of this study are available in the main text or Supplementary Information.

## Supplementary materials

Supplementary material 1 Molecular phylogenetic reconstruction of the Caucasian Hydrobiidae Data type jpg Explanation note **fig. S1**. Molecular phylogenetic reconstruction of the Caucasian Hydrobiidae based on the concatenated dataset (COI + 18S + 28S + H3). Genus-level clades of Caucasian taxa collapsed to a single branch. Branch of the new species *Gveleshapia kvevri* sp. nov. is in red color. A, Bayesian Inference, posterior probabilities higher than 0.95 are shown in the respective node. B, Maximum Likelihood, bootstrap values higher than 60% are shown in the respective nodes. Blue – Caucasopsiinae, violet – Bullaregiinae, yellow – Belgrandiellinae, green – Islamiinae (source Chertoprud et al. 2025). https://binary.pensoft.net/file/1558055This dataset is made available under the Open Database License (http://opendatacommons.org/licenses/odbl/1.0/). The Open Database License (ODbL) is a license agreement intended to allow users to freely share, modify, and use this Dataset while maintaining this same freedom for others, provided that the original source and author(s) are credited.Author: Elizaveta M. Chertoprud

Supplementary material 2 Phylogenetic tree Data type jpg https://binary.pensoft.net/file/1558056This dataset is made available under the Open Database License (http://opendatacommons.org/licenses/odbl/1.0/). The Open Database License (ODbL) is a license agreement intended to allow users to freely share, modify, and use this Dataset while maintaining this same freedom for others, provided that the original source and author(s) are credited.Author: Elizaveta M. Chertoprud

Supplementary material 3 Phylogenetic analyses Data type jpg https://binary.pensoft.net/file/1558057This dataset is made available under the Open Database License (http://opendatacommons.org/licenses/odbl/1.0/). The Open Database License (ODbL) is a license agreement intended to allow users to freely share, modify, and use this Dataset while maintaining this same freedom for others, provided that the original source and author(s) are credited.Author: Elizaveta M. Chertoprud

Supplementary material 4 Catalogue Data type xlsx Explanation note **table S1**. Examined material. https://binary.pensoft.net/file/1558058This dataset is made available under the Open Database License (http://opendatacommons.org/licenses/odbl/1.0/). The Open Database License (ODbL) is a license agreement intended to allow users to freely share, modify, and use this Dataset while maintaining this same freedom for others, provided that the original source and author(s) are credited.Author: Elizaveta M. Chertoprud

Supplementary material 5 Morphometry Data type xlsx Explanation note **table S2**. Shell measurements. https://binary.pensoft.net/file/1558059This dataset is made available under the Open Database License (http://opendatacommons.org/licenses/odbl/1.0/). The Open Database License (ODbL) is a license agreement intended to allow users to freely share, modify, and use this Dataset while maintaining this same freedom for others, provided that the original source and author(s) are credited.Author: Elizaveta M. Chertoprud
