# Supplementary figures and images for: Tiny guardian of the spring: Description of the first Islamiinae (Mollusca, Hydrobiidae) from the Caucasus
Source: Zookeys. 2026 Mar 10;1272:285–300. doi: 10.3897/zookeys.1272.178534 (PMC12997038; doi:10.3897/zookeys.1272.178534)

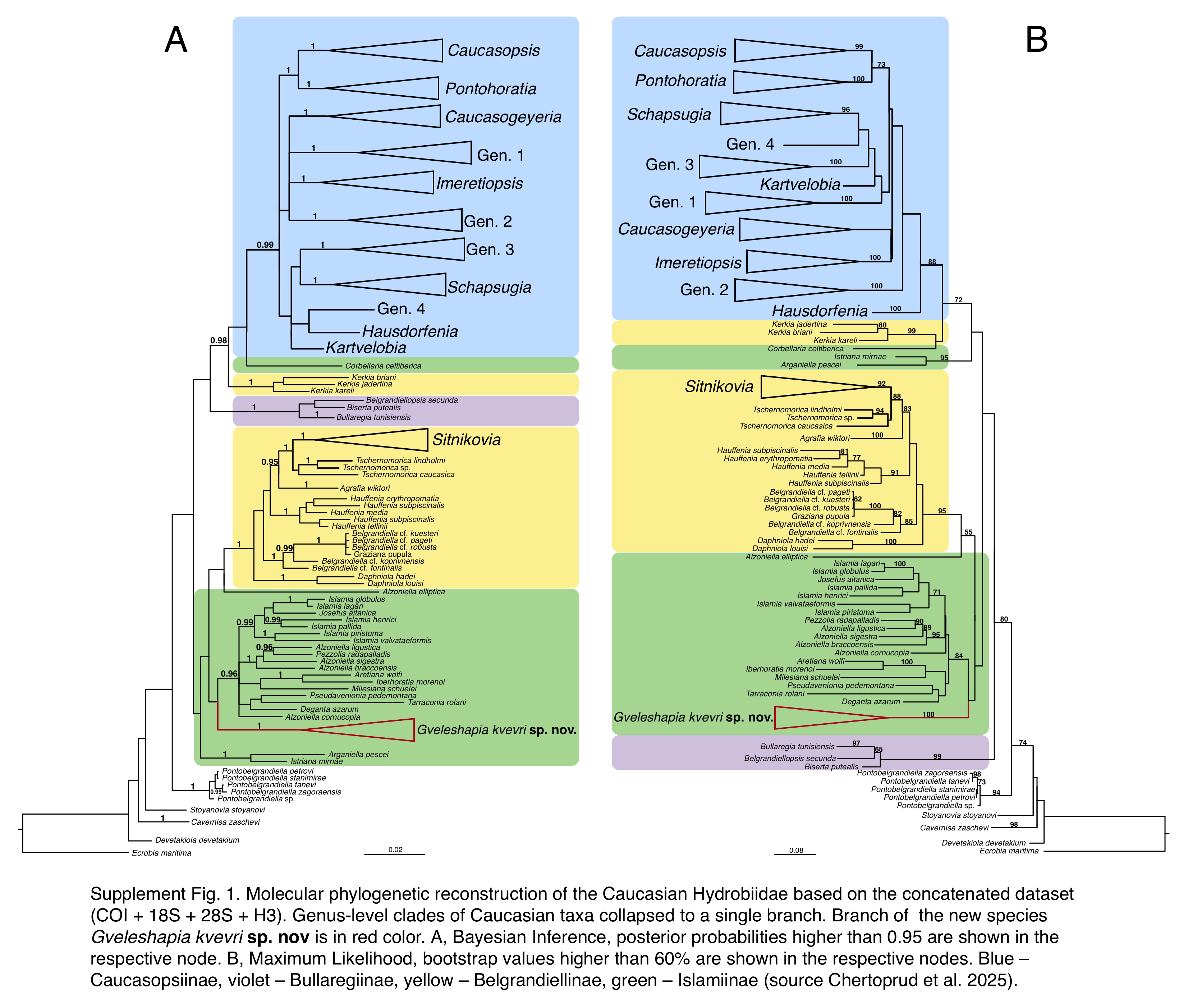

Supplement: Supplementary material 1 — Molecular phylogenetic reconstruction of the Caucasian Hydrobiidae [file zookeys-1272-285_article-178534__-s001.jpg]

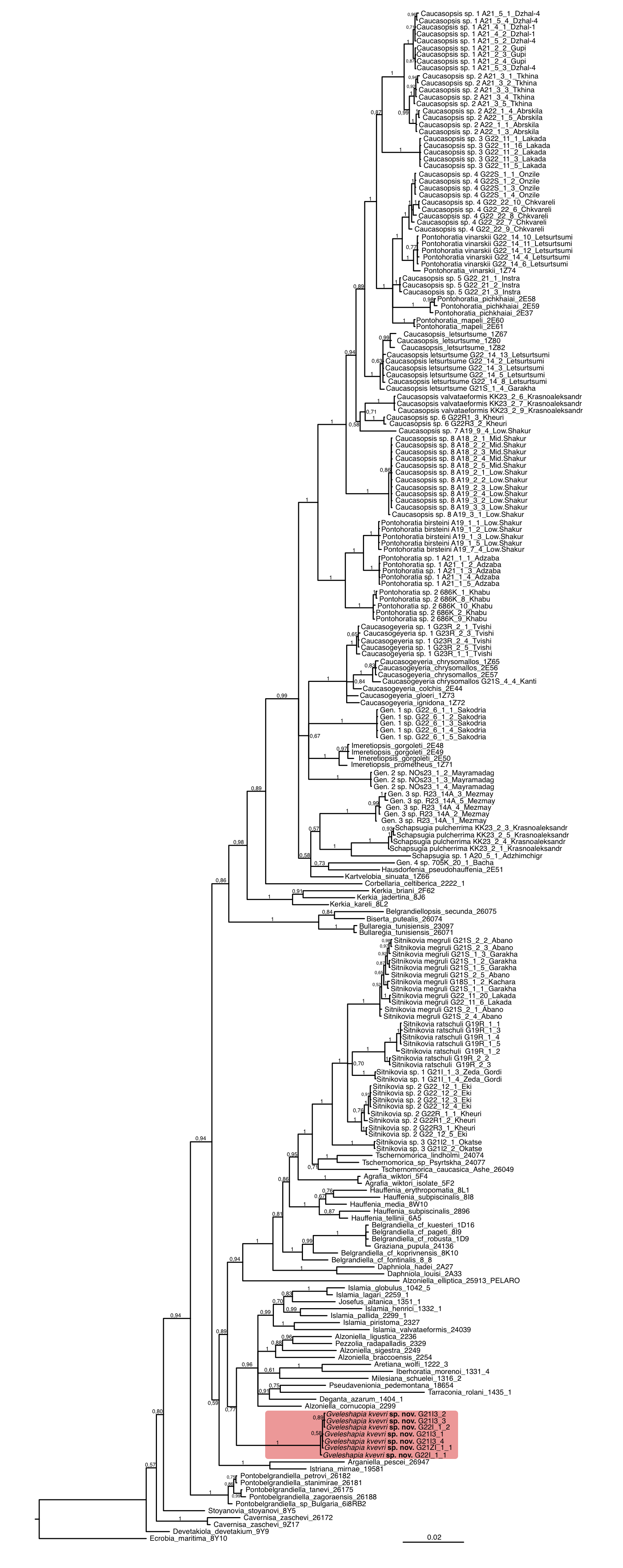

Supplement: Supplementary material 2 — Phylogenetic tree [file zookeys-1272-285_article-178534__-s002.jpg]

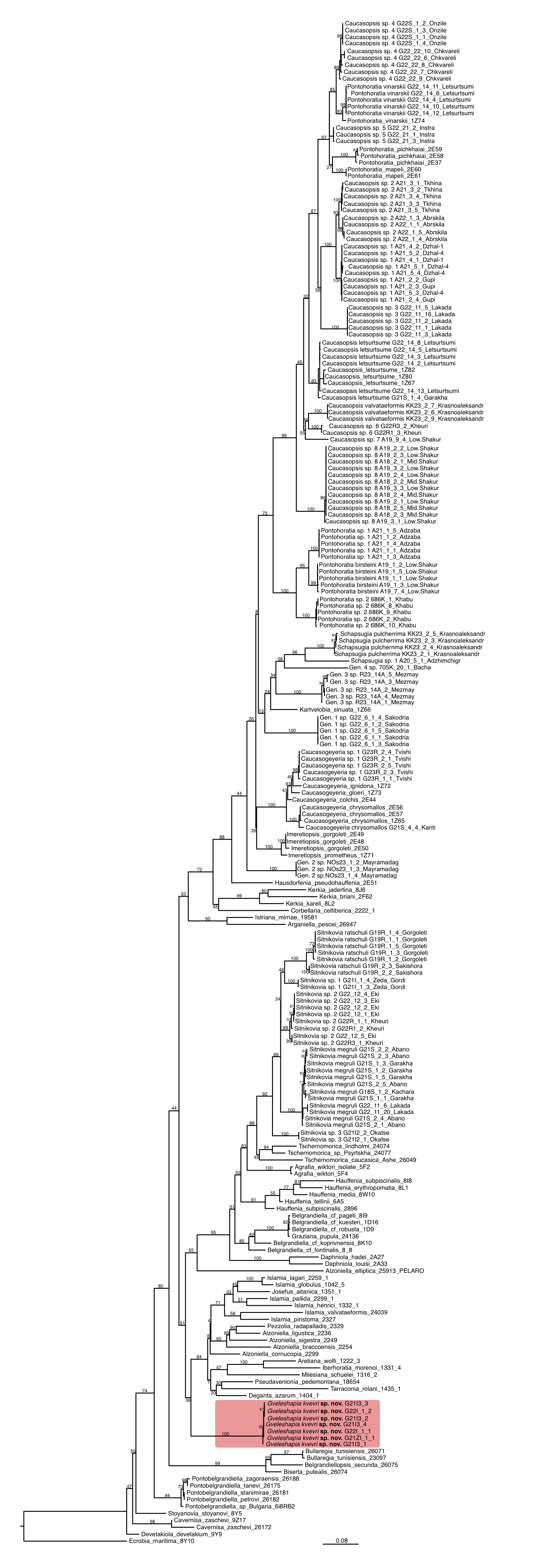

Supplement: Supplementary material 3 — Phylogenetic analyses [file zookeys-1272-285_article-178534__-s003.jpg]
